# Delineation of shear zone-hosted mineral targets within the Arabian-Nubian Shield, Egypt, using aeromagnetic data

**DOI:** 10.1038/s41598-026-45708-6

**Published:** 2026-04-17

**Authors:** Zeinab A. Shawky, Amin Esmail Khalil, Tark Arafa-Hamed, Rasha Tharwat, Alhussein Adham Basheer

**Affiliations:** 1 Geology Department, Faculty of Science, Capital University (formerly Helwan), 11790, Ain Helwan, Cairo, Egypt; 2 National Research Institute of Astronomy and Geophysics (NRIAG), Helwan, Cairo, 11421 Egypt

**Keywords:** Airborne magnetic survey, Arabian–Nubian Shield, Egyptian Eastern Desert, Structural interpretation, Mineral exploration, Planetary science, Solid Earth sciences

## Abstract

This study presents a pre-field airborne magnetic interpretation designed to delineate priority targets and guide subsequent ground-based exploration in the underexplored southern Eastern Desert of Egypt, a key sector of the Arabian-Nubian Shield. The analysis utilizes regional-scale airborne magnetic data (2-arc-minute grid resolution, acquired at ~4 km altitude) obtained from the National Oceanic and Atmospheric Administration (NOAA), and the National Centers for Environmental Information (NCEI) database. Multiple enhancement and interpretation techniques were applied to extract maximum structural information from the magnetic data. These included Centre for Exploration Targeting (CET) porphyry analysis, Source Edge Detection (SED) filtering, Euler deconvolution, and power spectral analysis, along with derivative filters (tilt, vertical, and horizontal gradients). Integration of these products enabled delineation of subsurface structures and estimation of depths to magnetic source ensembles. The interpretation reveals three major shear systems governing regional deformation: the interested Kharit-Hodein Megashear Zone (KHSZ), the Nugrus Shear Zone (NSZ), and the Allaqi-Heiani Shear Zone (AHSZ). These features, concentrated south of the Hafafit Core Complex (HCC), around Nugrus, and within the attentive Wadi Kharit depocenters, coincide with prominent magnetic anomalies and fault-controlled structures linked to the Upper Cretaceous Rift System. Euler deconvolution and power spectral analysis provide depth estimates to magnetic source ensembles, interpreted as lithological contacts, intrusive bodies, and basement structures, and magnetic source clusters ranging from near-surface to approximately 18 km, with quantified uncertainties of ±15–20% for spectral analysis and ±10–15% for Euler solutions. These depths represent centroids of magnetic source distributions, interpreted as crustal interfaces and intrusive complexes, rather than discrete body tops, and are consistent with regional crustal thickness estimates. Integration of magnetic anomaly patterns, structural lineaments, and porphyry signatures defines distinct high-, moderate-, and low-prospectivity zones. The convergence of high magnetic intensities and CET-derived porphyry signatures pinpoints the concerned Wadi Kharit depocenter and the Hafafit Core Complex margins as priority exploration targets for gold (orogenic and intrusion-related), copper (Volcanogenic Massive Sulfide** (**VMS) and porphyry-style), and rare earth element (REE) potential mineralization. This study establishes a data-driven predictive framework for subsequent ground validation and architecture, delineates structurally complex zones, and demonstrates the value of airborne magnetic surveys as a cost-effective first step for mineral exploration in complex shield terrains globally.

**Supplementary Information:**

The online version contains supplementary material available at 10.1038/s41598-026-45708-6.

## Introduction

Effective mineral exploration demands robust, data-driven targeting, de-risk, and focus costly field campaigns. This study provides the critical first step for the southern Eastern Desert of Egypt by processing and interpreting regional airborne magnetic data to generate a first-order prospectivity map, thereby identifying and ranking specific sites for future field validation, sampling, and detailed measurement.

**Fig. 1:**
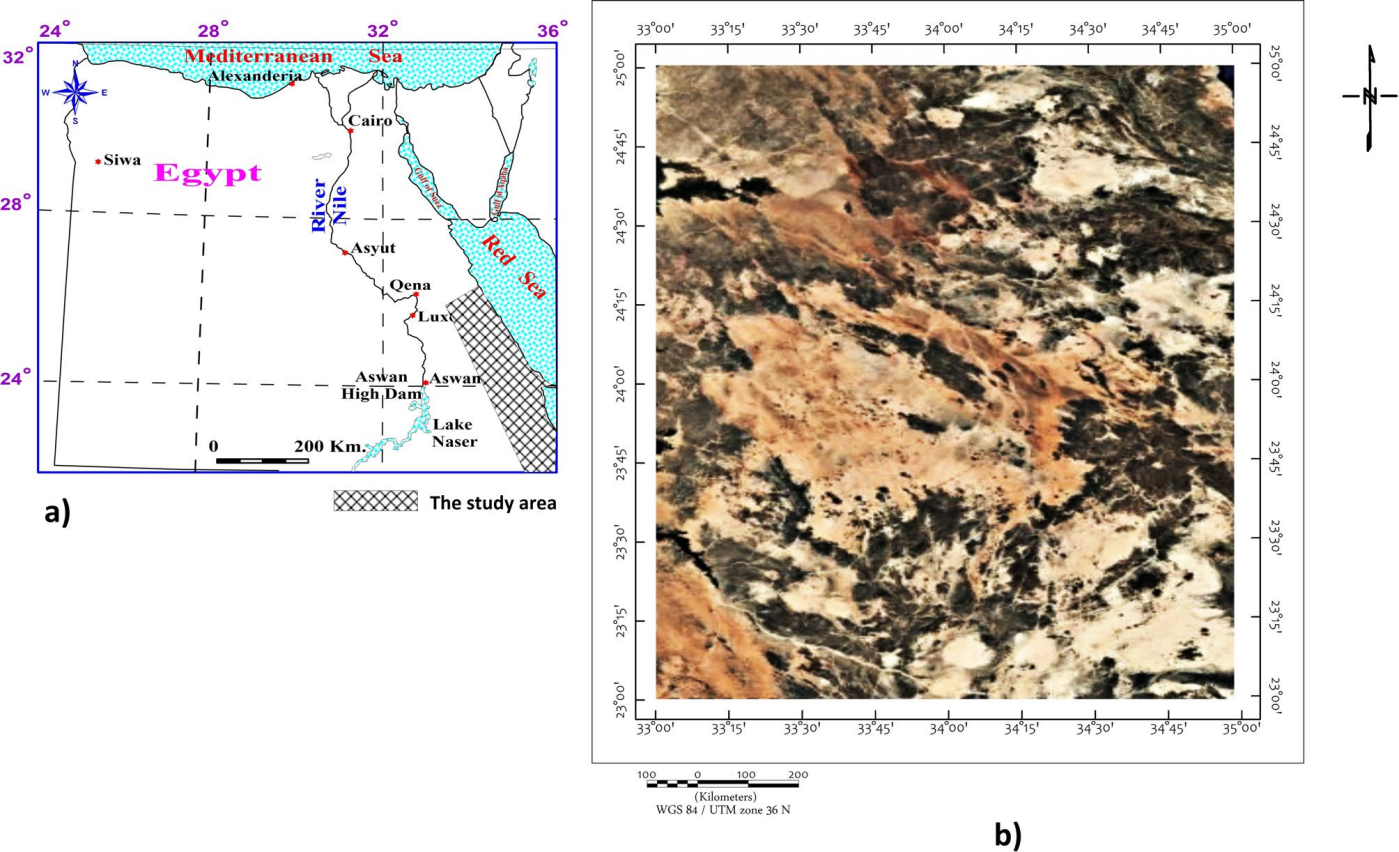
(**a**) Location map of Egypt showing the position of the study area (hatched zone) along the Eastern Desert between latitudes 23°–25°N and longitudes 33°–35°E, east of the River Nile and extending toward the Red Sea. Major cities and physiographic features such as Lake Nasser, Aswan High Dam, and the Red and Mediterranean Seas are indicated for reference.(**b**) Satellite image (WGS 84/UTM Zone 36N) illustrating the general geomorphological and lithological variations across the study area, highlighting the complex pattern of dark and light tonal contrasts that reflect differences in rock composition and structural fabric (Satalight Landsat Copernicus/IBCEO, https://earth.google.com/web).

The study area lies between latitudes 23° and 25° N and longitudes 33° and 35° E (Fig. 1), encompassing a strategically significant portion of the southern Eastern Desert, situated between the Nile Valley and the Red Sea. This region forms an integral part of the Arabian-Nubian Shield (ANS), a Proterozoic terrane renowned for its complex tectono-magmatic evolution and rich mineral endowment^1–3^. The area’s geological framework was shaped by successive episodes of accretion, deformation, and magmatism during the Pan-African orogeny^4–6^, producing a diverse lithological assemblage that includes ophiolitic remnants, metavolcanic belts, granitic intrusions, and high-grade metamorphic complexes such as gneiss, schist, and amphibolite^7–9^. These units are critical not only for reconstructing the geodynamic history of the ANS but also for their metallogenic significance, hosting economically valuable occurrences including orogenic gold, volcanogenic massive sulfide (VMS) deposits, and rare earth element (REE)-bearing pegmatites and granites^10,11^.

The investigated area exhibits an intricate array of structural trends, including fault systems that record crustal shortening and shearing, with sinistral strike-slip faults dominating the regional framework. In addition to these Proterozoic features, the study area hosts prominent Cretaceous basins, structurally controlled depressions within the Upper Egypt Rift System^12–16^. This rifting, accompanied by lithospheric thinning and crustal reactivation, has further enhanced the region’s mineralization potential by promoting fluid migration and remobilization of existing ore bodies. The structural complexity, particularly the prevalence of NW–SE and NNW–SSE sinistral shear zones, NE–SW extensional faults, and associated thrust systems, provides essential pathways for hydrothermal fluids and loci for mineral deposition^17,18^. Notable examples include the Nuqra and Kharit basins, structurally bounded depressions associated with the Upper Egypt Rift System that exhibit characteristics conducive to basin-hosted mineralization^19,20^.

This study specifically targets geological settings favorable for three deposit types: orogenic gold, volcanic-hosted massive sulfide (VMS) copper-zinc, and rare earth element (REE) mineralization associated with granitic intrusions and shear zone systems. The primary objectives are fourfold: first, to process and enhance regional airborne magnetic data using advanced filtering techniques, including tilt derivative (TDR), vertical derivative (VD), horizontal gradient (HG), CET (Center for Exploration Targeting) analysis, source edge detection (SED), and Euler deconvolution, to extract maximum structural information; second, to identify and characterize major structural lineaments (faults, shear zones, contacts) from magnetic derivatives and compare them with known geological structures; third, to integrate magnetic anomaly patterns, structural interpretations, and depth estimates to delineate prospective zones for gold, copper, and REE mineralization, thereby providing testable targets for subsequent field investigation; and finally, to contribute to Egypt’s national strategy for sustainable resource development by offering a scalable, non-invasive exploration methodology applicable to other regions within the Arabian-Nubian Shield and analogous terranes globally^21–23^.

Airborne magnetic surveys are essential for investigating subsurface structures in complex terrains like Egypt’s Eastern Desert, where limited surface exposure and multiple deformation phases often obscure direct geological observations. Magnetic data can penetrate this complexity to reveal hidden structural architecture. Our methodological approach integrates several complementary filtering techniques, each designed to enhance specific characteristics of magnetic data. Vertical and horizontal derivatives are first applied to amplify high-frequency signals from shallow sources, delineating near-surface contacts and fault traces^24–26^, while the tilt derivative normalizes vertical and horizontal gradients to balance responses from shallow and deep sources, thereby highlighting structural boundaries across varying depths^27,28^. Source edge detection further sharpens the boundaries of magnetic bodies, facilitating the mapping of lithological contacts and structural discontinuities^29,30^. Building on these enhancements, CET grid analysis identifies zones of structural complexity where multiple lineaments intersect, areas recognized as preferred sites for hydrothermal fluid flow and potential mineralization^31^. Concurrently, CET porphyry analysis detects circular magnetic patterns potentially associated with intrusive centers that may host porphyry-style copper-gold systems^32^. Finally, Euler deconvolution estimates depths to magnetic sources, enabling discrimination between shallow structures and deep-seated intrusions^33^. Together, these complementary techniques provide a robust framework for deciphering the subsurface architecture and delineating prospective zones within the study area.

By combining magnetic data interpretation with geological mapping and derivative processing techniques, this study not only enhances the understanding of subsurface architecture but also identifies new targets for mineral exploration^34^. The results successfully delineate potential mineralized zones, with the convergence of high magnetic intensities and distinct CET porphyry signatures, particularly within the Wadi Kharit region, defining them as priority exploration targets^35,36^. These findings contribute to an improved understanding of the subsurface mineral potential of the study area and provide a relevant basis for supporting sustainable resource management strategies, economic diversification, and the development of the mineral sector within national long-term planning frameworks.

## Geological setting

### Regional geological framework of the Arabian-Nubian Shield

The Eastern Desert is transected by an intense network of faults and shear zones that record a protracted tectonic history. For clarity in subsequent descriptions, we adopt the following terminology throughout this manuscript: a fault is defined as a discrete brittle fracture along which measurable displacement has occurred^37–39^; a shear zone represents a zone of deformation ranging from ductile to brittle-ductile behavior characterized by finite width^40^ a lineament refers to a linear feature identified in geophysical or remote sensing data that may correspond to underlying geological structures^41–44^; and a megashear zone denotes a crustal-scale shear zone typically exceeding 50 km in length, reflecting significant tectonic deformation^34,38,45^.

The Arabian-Nubian Shield (ANS) represents one of Earth’s largest exposures of juvenile Neoproterozoic crust, formed during the Pan-African orogeny (850-550 Ma) through accretion of intra-oceanic island arcs, oceanic plateaus, and microcontinental terranes^46,47^. This tectono-magmatic evolution accompanied the assembly of Gondwana, culminating in continent-continent collision along the East African Orogen.

The Neoproterozoic evolution of the ANS commenced with intra-oceanic subduction (c. 870-750 Ma), generating juvenile island arc assemblages including metavolcanics, metasediments, and associated plutonic suites. Subsequent terrane accretion (c. 750-620 Ma) involved oblique collision and suturing of these arcs along ophiolite-decorated suture zones, notably the Allaqi-Heiani, Yanbu, and Bir Umq sutures^46^. This accretionary phase was followed by the development of the Najd Fault System (c. 620-540 Ma), a crustal-scale transcurrent shear zone network extending >2000 km across the ANS. The Najd system comprises NW-trending sinistral strike-slip faults and associated ductile shear zones that accommodated late-orogenic escape tectonics and crustal extension^48^.

The ANS is endowed with diverse mineralization styles linked to its tectono-magmatic evolution. Regional metallogenic provinces include: (1) ophiolite-hosted chromite and nickel associated with suprasubduction zone mantle sequences; (2) volcanogenic massive sulfide (VMS) deposits within island arc assemblages; (3) orogenic gold mineralization concentrated along transcurrent shear zones and suture boundaries; and (4) rare metal mineralization (REE, Nb, Ta) within post-orogenic peralkaline granites and associated pegmatites^49–52^. This metallogenic framework provides the regional context for understanding mineralization controls within the Egyptian Eastern Desert sector of the ANS.

### Geology of the study area

The study area lies between latitudes 23° and 25° N and longitudes 33° and 35° E (Fig. 1), encompassing a strategically significant portion of the southern Eastern Desert of Egypt. This region extends from the Nile Valley in the west to the Red Sea coast in the east, spanning from Qena and Luxor in the north to Aswan in the south, and reaching eastward from the highlands near Hurghada toward the Halaib Triangle^53,54^.

The region has been subjected to multiple metamorphic events. Regional metamorphism, ranging from greenschist to amphibolite facies, is associated with the accretionary and collisional phases of the Pan-African orogeny, affecting the island arc and ophiolitic assemblages. This is overprinted by localized, high-temperature, low-pressure metamorphism related to the intrusion of syn- to post-orogenic granitoids. These metamorphic regimes are critical for mineralization, as greenschist facies conditions facilitate the formation of orogenic gold deposits, while contact metamorphism around intrusions can generate skarn-type mineralization and remobilize metals^50,55–57^.

**Fig. 2:**
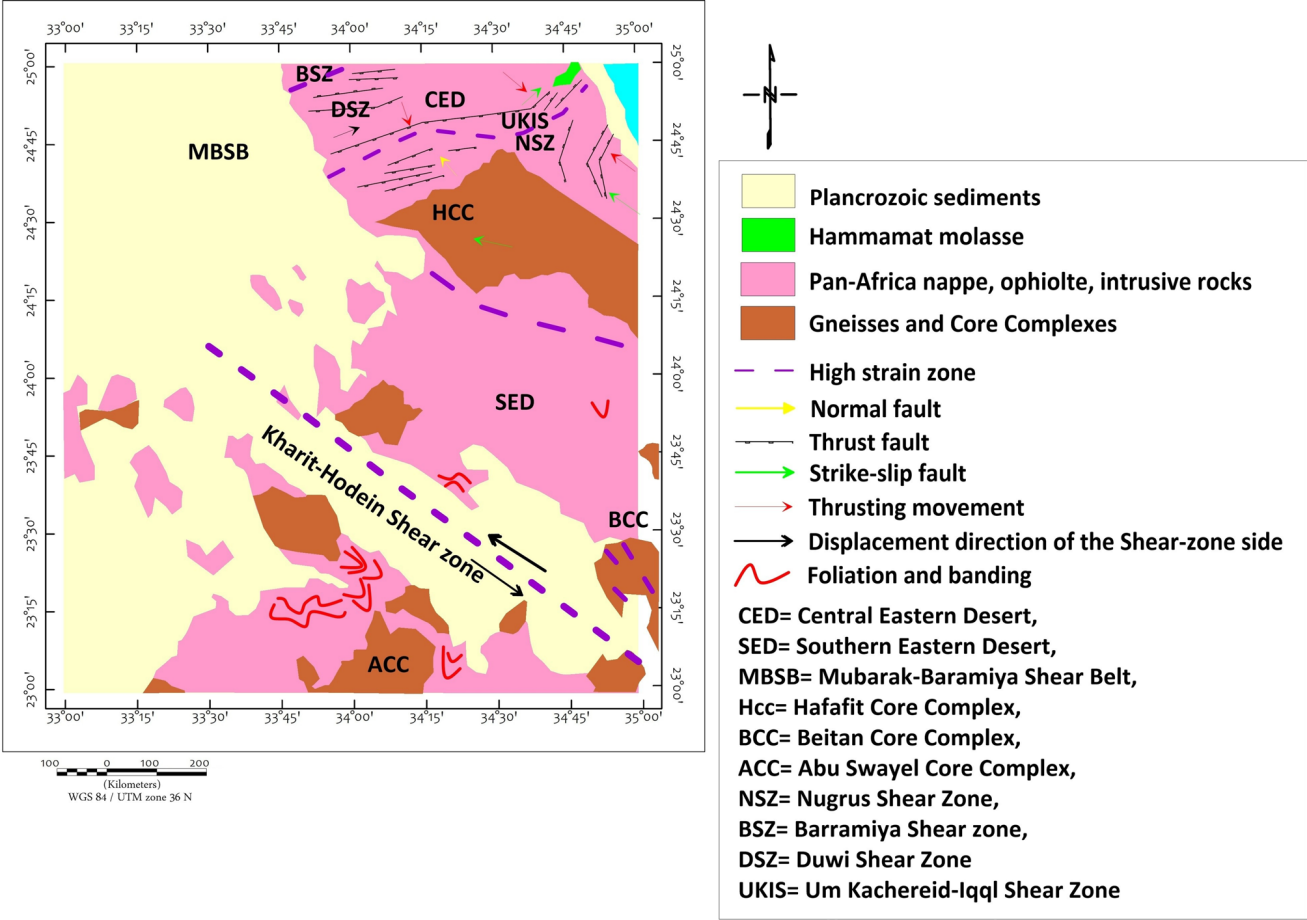
Structural map of the study area shows the major structural features modified after^5^, CED= Central Eastern Desert, SED= Southern Eastern Desert, MBSB= Mubarak-Baramiya Shear Belt, Hcc= Hafafit Core Complex, UKIS= Um Kachereid-Iqql Shear Zone, BCC= Beitan Core Complex, ACC= Abu Swayel Core Complex, NSZ= Nugrus Shear Zone, BSZ= Barramiya Shear zone, DSZ= Duwi Shear Zone, Using ArcGIS 10.2.2, https://enterprise.arcgis.com/en/inspire/10.3/get-started/release-notes-10-2-1-for-inspire.htm).

**Fig. 3:**
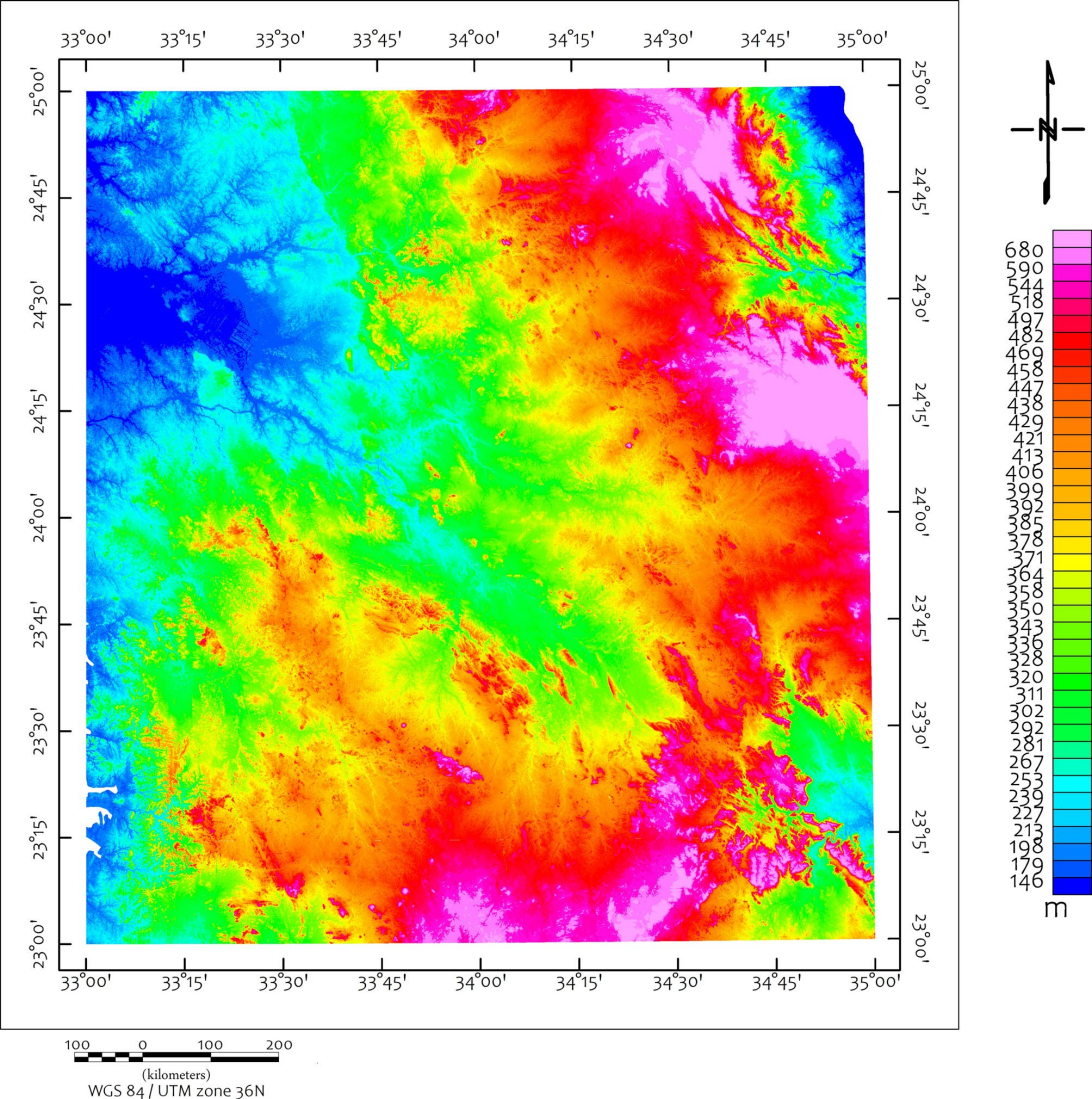
DEM map of the study area.

The study area exposes a complete spectrum of ANS lithologies, reflecting its complex tectono-magmatic evolution. Ophiolitic assemblages dominate the basement complex, comprising serpentinized peridotites, gabbros, sheeted dykes, and pillowed basalts representing obducted Neoproterozoic oceanic lithosphere^46,57–60^. These units are particularly well-exposed in the Wadi Ghadir and Wadi Kharit sectors (Fig. 2). The terrain is rugged with elevations ranging from 145 m to 680 m above sea level (Fig. 3).

Island arc metavolcanic sequences include metamorphosed basaltic-andesitic lavas, pyroclastics, and intercalated metasediments, recording intra-oceanic subduction processes. These assemblages host VMS-style mineralization and are extensively developed in the central and southern parts of the area^61^.

Syn- to post-orogenic granitoids intrude the older basement, ranging from syn-tectonic tonalites and granodiorites (c. 650-610 Ma) to post-tectonic alkali feldspar granites (c. 600-550 Ma). The latter include peralkaline and highly fractionated varieties enriched in rare earth elements (REEs), niobium, and tantalum, particularly within the Hafafit and Nugrus areas^51,52,62^.

Gneissic core complexes, exemplified by the Hafafit Core Complex (HCC) in the northeastern sector, expose high-grade metamorphic rocks (gneisses, schists, amphibolites) exhumed during late-orogenic extension and core complex formation^9^. The HCC is structurally bounded by major shear zones and thrust systems, creating a tectonically complex terrain favorable for fluid focusing and potential mineralization.

The structural framework is dominated by NW- to NNW-trending sinistral shear zones, NE-SW extensional faults, and associated thrust systems. The Kharit-Hodein Megashear Zone (KHSZ) is a crustal-scale ductile shear zone extending for nearly 100 km in a NW direction, constituting a major tectonic boundary that governs the regional structural architecture^17^. Its pronounced lateral displacement and ductile deformation fabrics indicate significant left-lateral movement controlling the positioning and evolution of adjacent structural basins.

The Nugrus Shear Zone (NSZ) demarcates the boundary between the Wadi Ghadir ophiolites and the Hafafit Core Complex in the northeastern part of the study area. This structure plays a critical role in controlling deformation and fluid pathways along the HCC margin^18^. The Allaqi-Heiani Suture represents a major Pan-African suture zone preserving remnants of Neoproterozoic oceanic lithosphere and marking the boundary between accreted terranes. Although its main trace lies south of the study area, its structural influence extends into the region, with subsidiary splays contributing to the overall tectonic complexity^50^.

Superimposed on the Precambrian basement are Cretaceous extensional basins related to the Upper Egypt Rift System^13^. The Nuqra Basin exhibits a fault-controlled, asymmetric half-graben geometry shaped by NW- and N-S trending faults, with steeply east-dipping strata indicating progressive fault growth and tilting during rifting. The Kharit Basin, situated adjacent to the KHSZ, displays a NE-dipping structural geometry with twin depocenters defined by fault systems genetically linked to the KHSZ. These basins record differential subsidence and deformation controlled by oblique-slip reactivation along pre-existing shear fabrics^19,20^.

The study area hosts diverse potential mineralization styles reflecting its complex geological history. Orogenic gold occurs in quartz veins within ophiolitic and granitic rocks along major shear zones, with significant occurrences at Hamash and analogues to the world-class Sukari deposit located further north^63–65^. VMS copper-zinc mineralization is associated with island arc metavolcanic sequences, particularly in the southern sectors^61^. REE-bearing granites and associated pegmatites within post-orogenic intrusions, especially in the Hafafit and Nugrus areas, show elevated rare earth element concentrations of economic interest^52^. Additionally, sedimentary phosphorites of Cretaceous-Paleogene age occur along the Red Sea coastal plains near Safaga and Quseir, contributing to Egypt’s agricultural resource base^66^. This diversity of potential mineralization styles, combined with the region’s structural complexity, establishes the southern Eastern Desert as a priority target for integrated geophysical exploration.

## Methodology

### Data acquisition

Airborne magnetic data were obtained from the National Centers for Environmental Information (NCEI) database^67^, representing a regional compilation of surveys across the Egyptian Eastern Desert. This data comprises a regional 2-arc-minute grid, equivalent to approximately 3.7 km × 3.7 km cell-size at the equator. These grids were compiled from multiple airborne surveys conducted between 1960 and 1990, flown at varying altitudes with an average of approximately 4 km above terrain. While this altitude limits sensitivity to short-wavelength anomalies from shallow sources, subsequent enhancement filters were applied to extract maximum structural information from the available data. Although this resolution reflects a regional-scale dataset, rather than ultra-detailed local acquisition, the data were carefully reprocessed and enhanced to achieve high-resolution interpretive capability. The term ‘high-resolution’ describes interpretive products derived from enhancement filtering of regional-scale magnetic data (2-arc-minute grid, ~4 km altitude), not the original survey resolution. The original magnetic measurements were corrected for external field effects by subtracting the Comprehensive Model CM4^68,69^, ensuring magnetic field accuracy and internal consistency. Reduction to Pole (RTP) was applied using the Geosoft Oasis Montaj implementation with the following parameters: Inclination = 37.5° (IGRF-13 for the study area center, 24°N, 34°E), Declination = 2.3°, assuming induced magnetization dominates over remanent components. This assumption is reasonable given the predominantly magnetite-bearing basement lithologies. Line-leveling procedures were subsequently applied to minimize crossover errors and improve internal continuity between flight lines^70^.

While the initial dataset is consistent with a regional compilation, the application of a suite of advanced derivative and edge-detection filters, including the Tilt Derivative (TDR), Vertical Derivative (VD), Horizontal Gradient (HG), Center for Exploration Targeting (CET) analyses, and Source Edge Detection (SED), significantly enhanced the local signal content. These filters accentuate short-wavelength, high-frequency components of the magnetic field, thereby isolating and resolving near-surface and structurally controlled magnetic features. This multi-filter enhancement effectively transforms the regional grid into a dataset capable of supporting detailed structural interpretation, revealing subtle discontinuities and fault-bounded zones that define potential sites of mineralization. Therefore, the incorporation of these supports the interpretation that magnetic products justifies the use of the term “high resolution” to refer to the improved interpretation results rather than to the acquisition criteria, even though the primary data are derived from a wider and more regional survey in nature (two-minute arc grid, elevation of about 4 km)^65^.

### Data processing

Data processing for airborne magnetic survey involves refining raw data into meaningful magnetic anomaly maps, isolating signals associated with mineral deposits from background noise. This was achieved using horizontal gradient, tilt derivative, vertical derivative, and theta map filters to retain signals relevant to geological formations^29,64^. To enhance deeper magnetic sources and structural features, transformation techniques such as spectral analysis, CET grid analysis, CET porphyry analysis, source edge detection, and Euler deconvolution were applied^71–74^. All data processing and interpretation were performed using^75,76^and^77^.

The interpretation combined qualitative and quantitative analyses to infer mineral deposits. Spectral analysis, CET grid analysis, source edge detection, and CET porphyry analysis enabled visualization of magnetic anomaly depth and orientation^78–80^. These techniques facilitated detailed examination of magnetic sources (e.g., ophiolitic sequences, granitic intrusions, fault-associated deposits). Quantitative anomaly trend analysis tracked spatial orientation and continuity of magnetic features, identifying structural trends like faults/shear zones that host potential mineralization^80^. This integrated approach mapped geologic structures and magnetic signatures indicative of valuable minerals, highlighting zones for exploration drilling.

The interpretation followed a sequential processing workflow designed to extract maximum structural information from the airborne magnetic data. Spectral analysis (supplementary material) first estimated average depths to magnetic sources and guided filter selection. The vertical derivative, horizontal gradient, tilt derivative (TDR), and theta map filters were sequentially applied to delineate near-surface contacts, fault traces, and structural boundaries across varying depths. Source edge detection (SED) subsequently sharpened the boundaries of magnetic bodies, enabling precise mapping of intrusive contacts and shear zone margins. The Center for Exploration Targeting Grid-Analysis (CET-GA) technique then identified zones of high lineament density and intersection, critical pathways for hydrothermal fluid flow, while CET Porphyry Analysis detected circular magnetic patterns characteristic of intrusive centers potentially hosting porphyry-style copper-gold systems. Finally, Euler deconvolution provided depth estimates to magnetic sources, discriminating between shallow structures and deep-seated intrusions. This integrated framework, with each technique cross validating the others, enabled a coherent reconstruction of subsurface architecture and a robust predictive model of mineral potential.

## Results

**Fig. 4:**
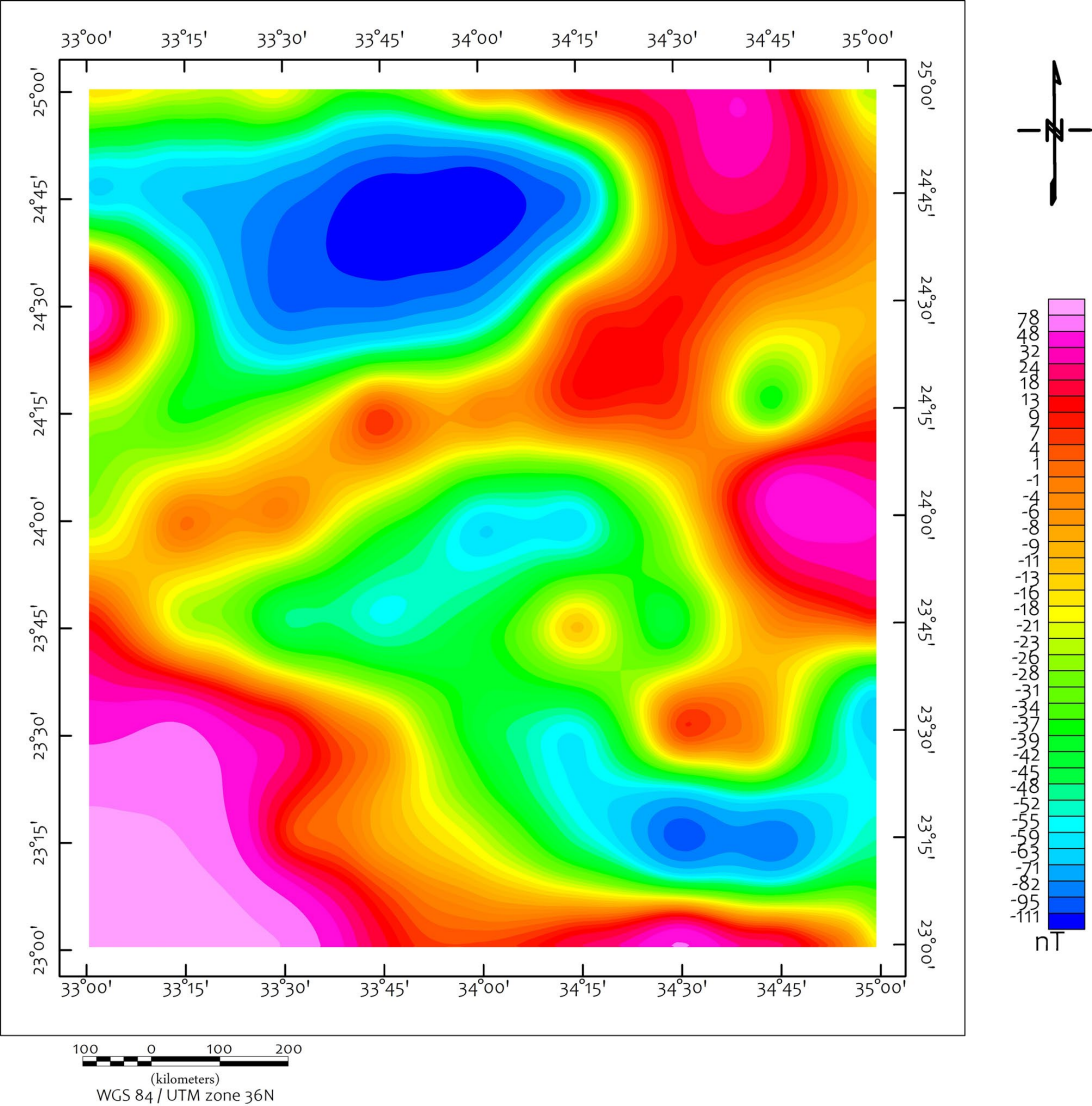
The RTP map of the utilized aeromagnetic data, Basic source data from https://www.ncei.noaa.gov/products/earth-magnetic-model-anomaly-grid-2. Using Geosoft Oasis Montaj 2015 v. 8.4 software, https://www.seequent.com/help-support/oasis-montaj/).

The derived structural and lithological maps. pinpoint several high-priority anomalies and prospective zones, which are proposed as optimal locations for initial ground-truthing, rock sampling, and infill geophysical surveys. The airborne magnetic data acquired over the southern Eastern Desert of Egypt have delineated a complex subsurface architecture marked by distinct structural, lithological, and magnetic signatures indicative of mineral potential. The RTP magnetic anomaly map (Fig. 4) reveals several distinct magnetic domains that correlate with known lithological units. High-amplitude, short-wavelength anomalies (1-70 nT in Fig. 4) correspond to outcropping and near-surface ophiolitic assemblages (serpentinites, gabbros) with high magnetite content, particularly in the central and eastern sectors. In contrast, broad, low-amplitude magnetic lows (-123:-21 n.T in Fig. 4) coincide with granitic intrusions and gneissic terrains of the Hafafit Core Complex, reflecting their relatively low magnetic susceptibility. Superimposed on these domains, linear magnetic gradients, abrupt transitions between magnetic highs and lows, align with major fault systems, notably the NW-trending Kharit-Hodein Megashear Zone, indicating structural juxtaposition of contrasting lithologies along these tectonic boundaries. These variations in magnetic intensity reflect contrasting rock susceptibilities, enabling the differentiation of subsurface structures associated with potential mineralization zones.

**Fig. 5:**
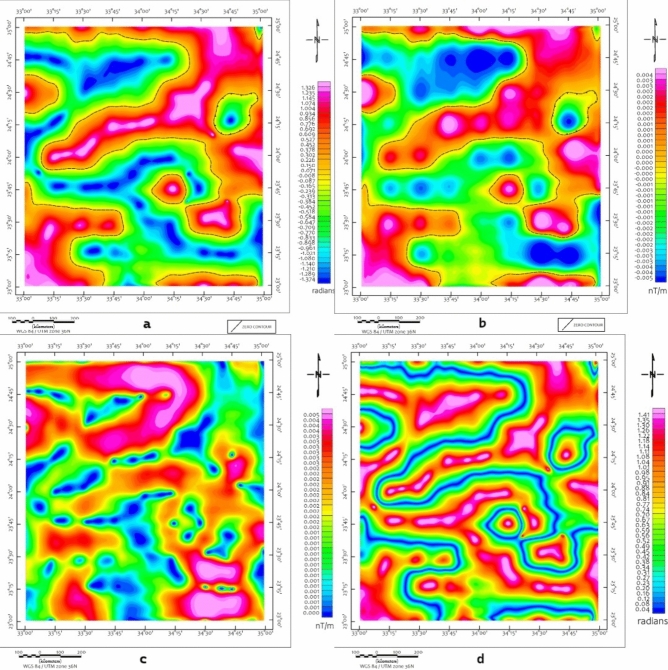
(**a**) TD, (**b**) VD, (**c**) HG and (**d**) TM of the RTP map (Using Geosoft Oasis Montaj 2015 v. 8.4 software, https://www.seequent.com/help-support/oasis-montaj/).

A suite of edge-detection and structural enhancement filters (Fig. 5), Total Derivative (TD), Vertical Derivative (VD), Horizontal Gradient (HG), and Tilt Derivative (TM) was applied to the RTP data. These filtered maps reveal well-defined linear magnetic anomalies trending predominantly NW to NNW, consistent with the dominant sinistral strike-slip fault systems observed in surface geology. The directional consistency between geophysical lineaments and mapped faults (Fig. 2) supports their structural continuity at depth, consistent with observations from similar ANS terrains^22,81^.

**Fig. 6:**
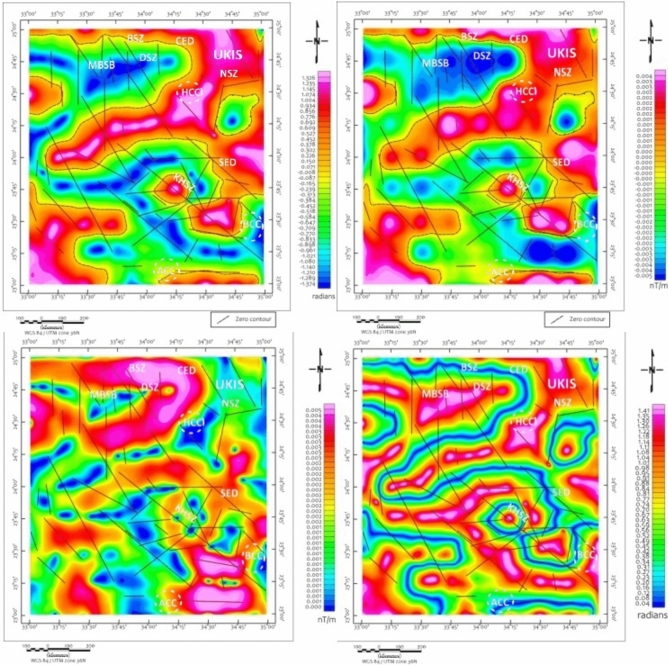
CET-GA of maps (**a**) TD, (**b**) VD, (**c**) HG and (**d**) TM of the RTP map, Brief terms: KHSZ= Kharit-Hodein Shear Zone, CED= Central Eastern Desert, SED= Southern Eastern Desert, MBSB= Mubarak-Baramiya Shear Belt, Hcc= Hafafit Core Complex, BCC= Beitan Core Complex, ACC= Abu Swayel Core Complex, NSZ= Nugrus Shear Zone, BSZ= Barramiya Shear zone, DSZ= Duwi Shear Zone, UKIS= Um Kachereid-Iqql Shear Zone, (Using Geosoft Oasis Montaj 2015 v. 8.4 software, https://www.seequent.com/help-support/oasis-montaj/).

**Fig. 7:**
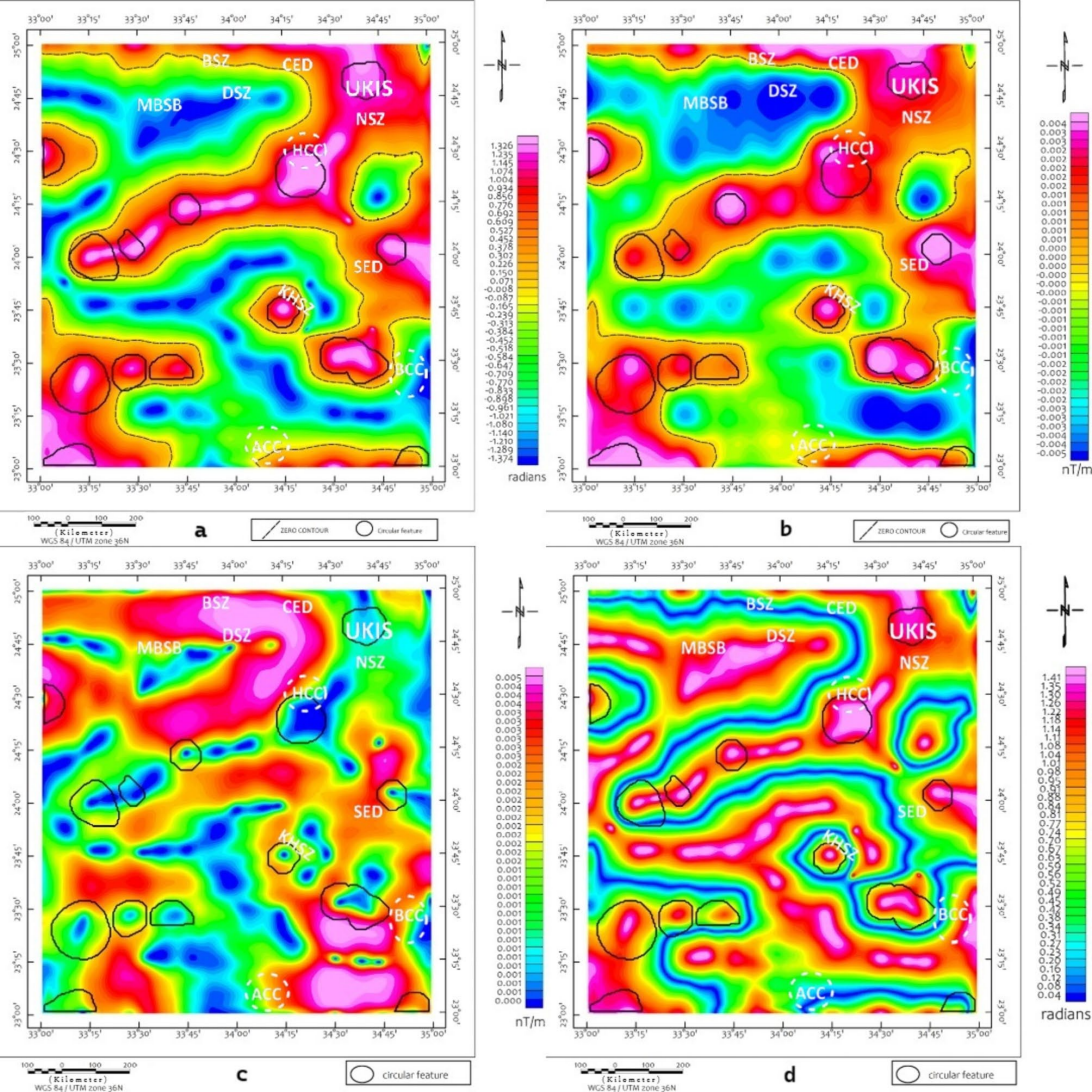
CET porphyry analysis superimposed on (**a**) TD, (**b**) VD, (**c**) HG and (**d**) TM of the RTP map , Brief terms: KHSZ= Kharit-Hodein Shear Zone, CED= Central Eastern Desert, SED= Southern Eastern Desert, MBSB= Mubarak-Baramiya Shear Belt, Hcc= Hafafit Core Complex, BCC= Beitan Core Complex, ACC= Abu Swayel Core Complex, NSZ= Nugrus Shear Zone, BSZ= Barramiya Shear zone, DSZ= Duwi Shear Zone, UKIS= Um Kachereid-Iqql Shear Zone, (Using Geosoft Oasis Montaj 2015 v. 8.4 software, https://www.seequent.com/help-support/oasis-montaj/).

CET edge-detection and porphyry analysis maps (Figs. 6 and 7) provide enhanced resolution of structural complexity, particularly in regions exhibiting clustered circular magnetic features. These patterns, especially prominent in the southern and central blocks, are suggestive of intrusive bodies, though confirmation requires ground investigation. These features, especially prominent in the southern and central blocks, are suggestive of intrusive bodies or porphyry systems. The CET-GA results highlight several geophysically distinct zones, particularly around the Wadi Kharit region, where concentric circular magnetic signatures are observed. Such patterns are commonly associated with intrusive centers in various geological settings globally^73–75^. although magnetic data alone cannot definitively establish the presence of porphyry-style potential mineralization. These circular features, combined with elevated magnetic gradients and structural complexity, suggest potential intrusive bodies that warrant ground investigation. These signatures correlate spatially with mapped shear zones and thrust-related structures, suggesting structurally focused zones of hydrothermal alteration and mineral deposition.

**Fig. 8:**
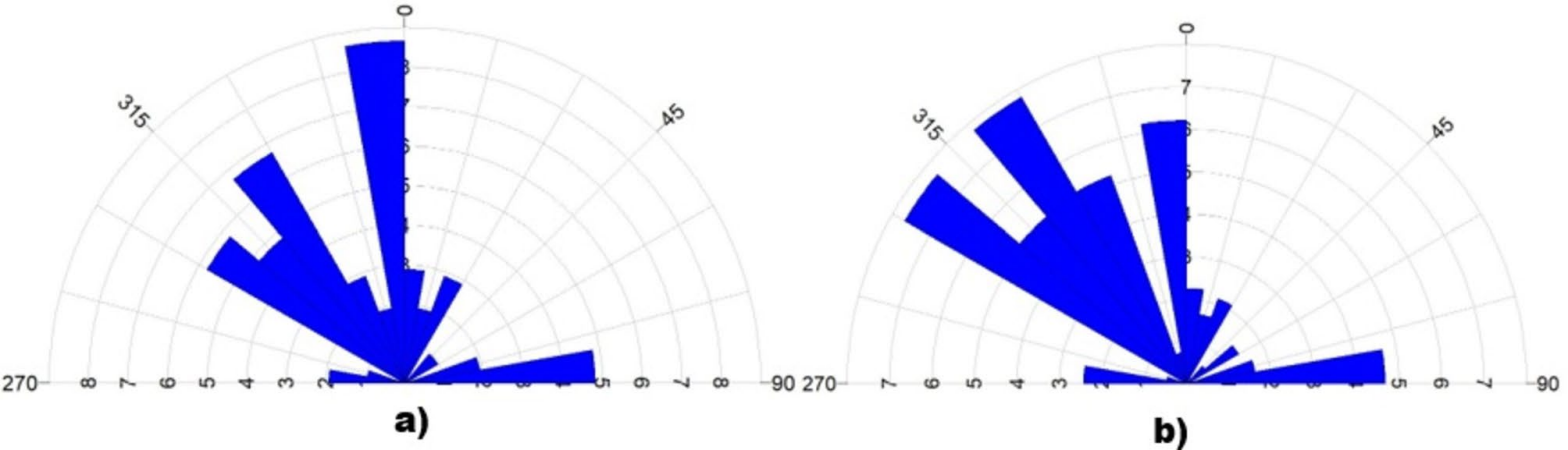
Rose diagram showing (**a**) the structural trends interpreted from aeromagnetic data هncluding both shallow and deep structures, (**b**) the lineament length (Using RockWare Inc. RockWorks v 16. RockWare Inc., https://www.rockware.com/product/rockworks/).

Rose diagram analysis of magnetic lineaments (Fig. 8a) confirms a dominant NW structural trend, with subordinate N–S and NE orientations. The lineament length distribution (Fig. 8b) reveals that the longest and most continuous structural features delineate the boundaries of the Kharit–Hodein Megashear Zone (KHSZ) and the Nugrus Shear Zone (NSZ), both exhibiting pronounced magnetic signatures with lateral continuity exceeding tens of kilometers. This coherent NW–NNW structural trend traced across the study area is consistent with known crustal-scale shear corridors controlling regional deformation in the Arabian–Nubian Shield^82^, validating the interpretation of the KHSZ as a first-order tectonic boundary governing major lithological and structural architecture in the southern Eastern Desert.

**Fig. 9:**
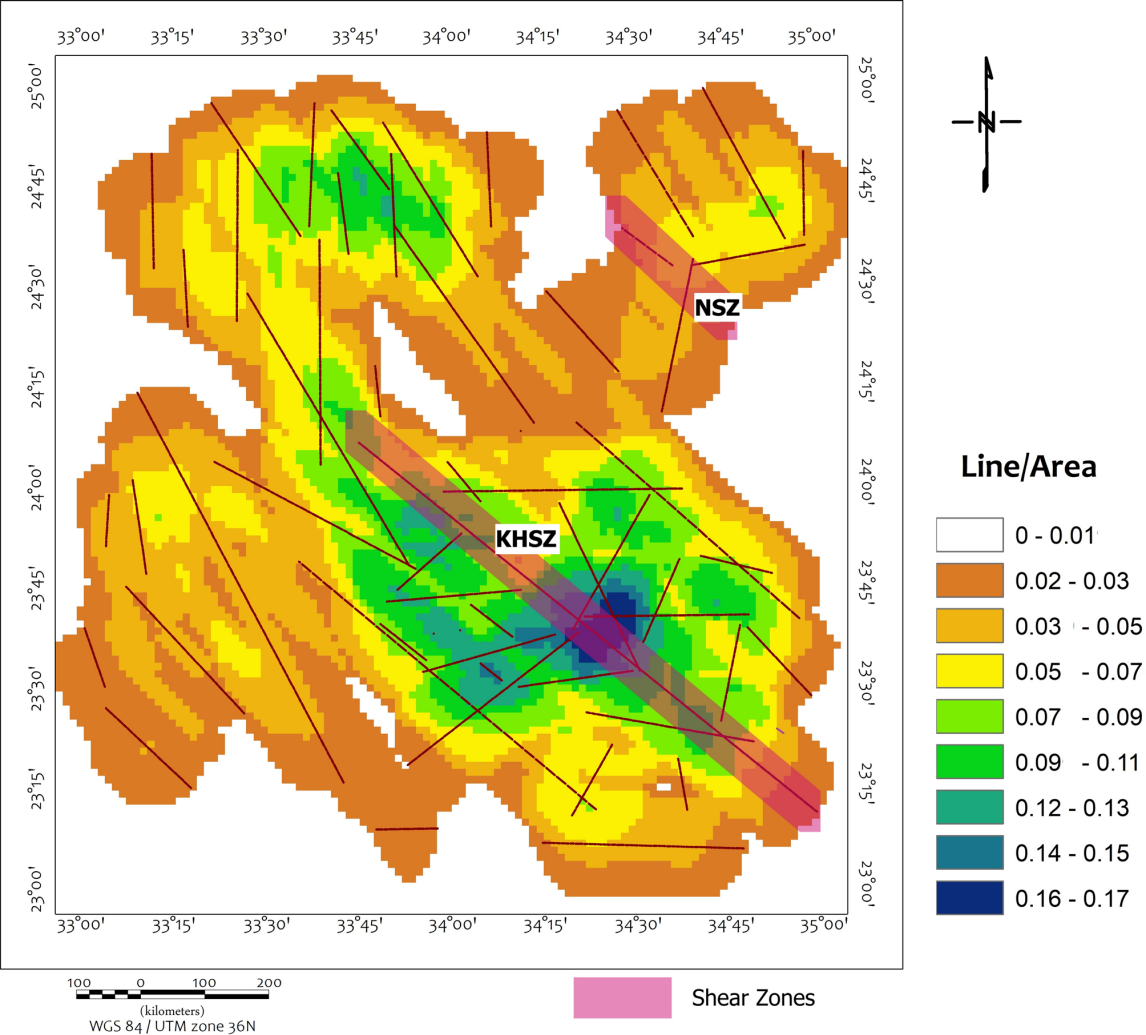
Density map with the structural lineaments highlight two major shear zones in the area, KHSZ= Kharit-Hodein Shear Zone, NSZ= Nugrus Shear Zone (Using Esri. ArcMap (Using ArcGIS 10.2.2, https://enterprise.arcgis.com/en/inspire/10.3/get-started/release-notes-10-2-1-for-inspire.htm).

The lineament density map (Fig. 9) provides quantitative insights into crustal deformation patterns, highlighting the spatial concentration and orientation of structural features. Areas of high lineament density (0.6–1.0 km/km^2^) occur prominently within the Wadi Kharit region and the southwestern quadrant, corresponding closely with regions of elevated magnetic gradients and coinciding with the positions of interpreted intrusive bodies. These high-density zones suggest that structural reactivation has enhanced crustal permeability, facilitating magmatic and hydrothermal activity. This reactivation is primarily attributed to the Cretaceous extensional event associated with the Upper Egypt Rift System, which rejuvenated pre-existing Pan-African shear fabrics including the KHSZ and NSZ.

The KHSZ is clearly delineated as a continuous NW–NNW-trending belt of high lineament density extending across the central part of the map, reflecting its role as a first-order crustal shear corridor. To the northeast, the NSZ forms a parallel, moderately dense structural band (0.4–0.6 km/km^2^) that demarcates the boundary of the Hafafit Core Complex. Additional subsidiary structural lineaments trending NE–SW and E–W, interpreted as splays or subsidiary shear zones based on their coherent magnetic signatures and intersection geometry, intersect both megashears, generating clusters of structural intersections that mark zones of enhanced deformation and potential fluid flow.

Collectively, the lineament density distribution defines the tectonic grain of the southern Eastern Desert, characterized by high-density NW–NNW trending lineaments as confirmed by rose diagram analysis. This pattern highlights the structural control exerted by the KHSZ, NSZ, and their associated plays on crustal architecture and potential mineralization pathways, providing a coherent framework for understanding fluid flow and targeting prospective zones within the study area.

**Fig. 10:**
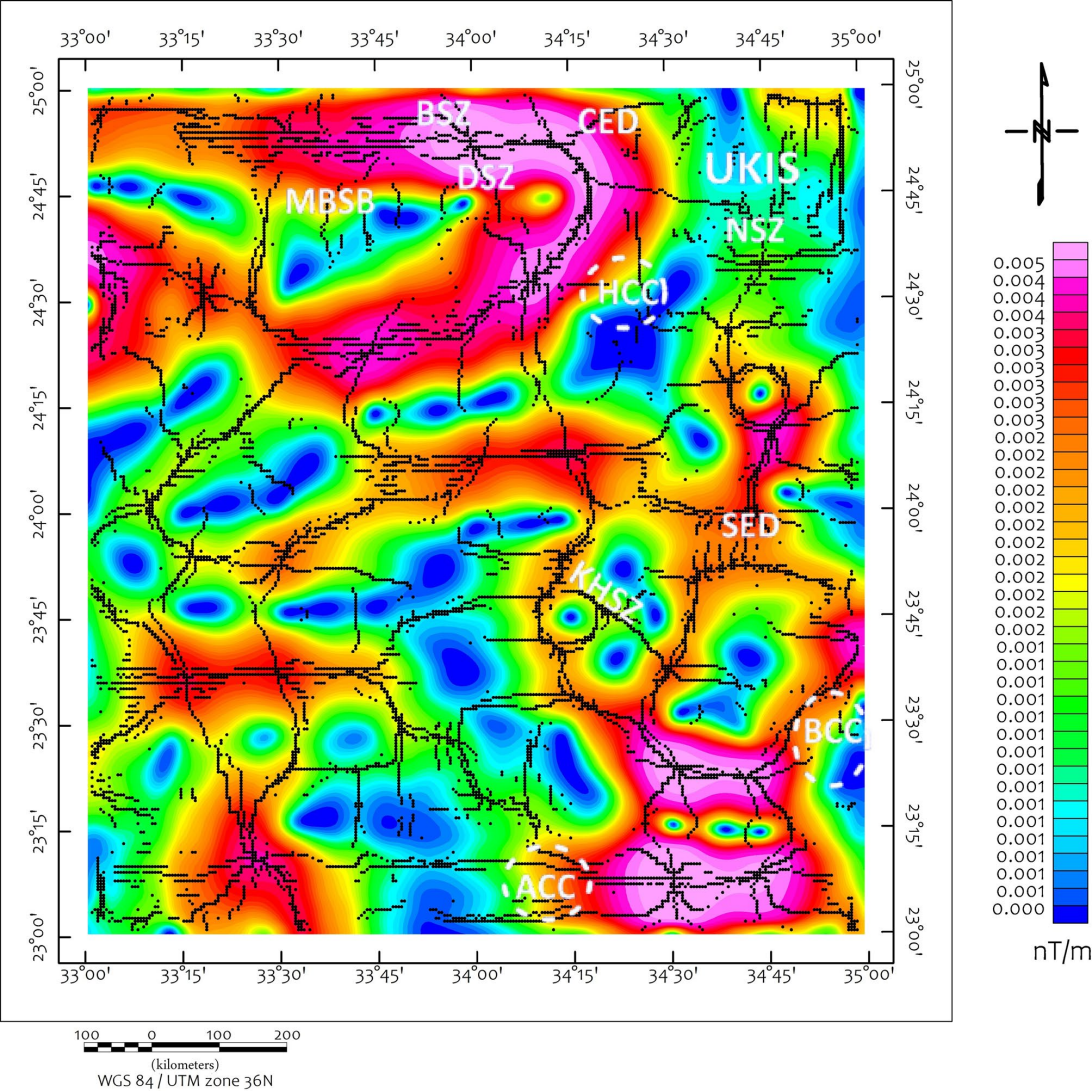
SED map of the horizontal gradient, Brief terms: KHSZ= Kharit-Hodein Shear Zone, CED= Central Eastern Desert, SED= Southern Eastern Desert, MBSB= Mubarak-Baramiya Shear Belt, Hcc= Hafafit Core Complex, BCC= Beitan Core Complex, ACC= Abu Swayel Core Complex, NSZ= Nugrus Shear Zone, BSZ= Barramiya Shear zone, DSZ= Duwi Shear Zone, UKIS= Um Kachereid-Iqql Shear Zone.(Using Geosoft Oasis Montaj 2015 v. 8.4 software, https://www.seequent.com/help-support/oasis-montaj/).

**Fig. 11:**
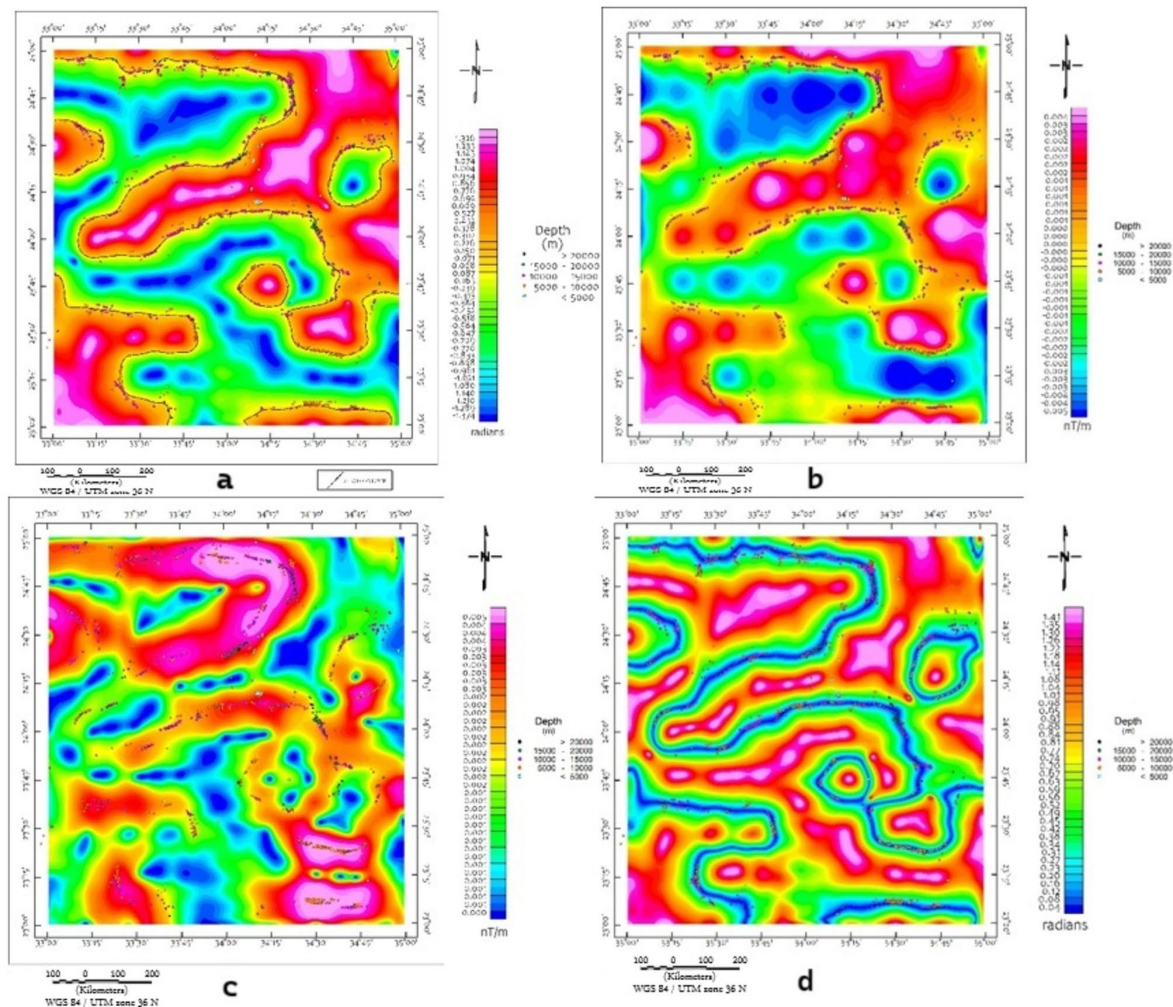
Euler deconvolution of SI=0 superimposed on (**a**) TD, (**b**) VD, (**c**) HG and (**d**) TM of the RTP map (Using Geosoft Oasis Montaj 2015 v. 8.4 software, https://www.seequent.com/help-support/oasis-montaj/).

The Structural Edge Detection (SED) map (Fig. 10) delineates discrete zones of structural discontinuities, which coincide with the magnetic anomalies and align with the Euler deconvolution results (Fig. 11). These match remarkably well with the CET-GA outputs, supporting the identification of key geological contacts and possible mineralized zones. Depth estimation from Euler deconvolution, constrained to a structural index of 0, suggests that these contacts range from near-surface expressions to depths exceeding 10 km, particularly in areas with dense clustering of solutions.

**Fig. 12:**
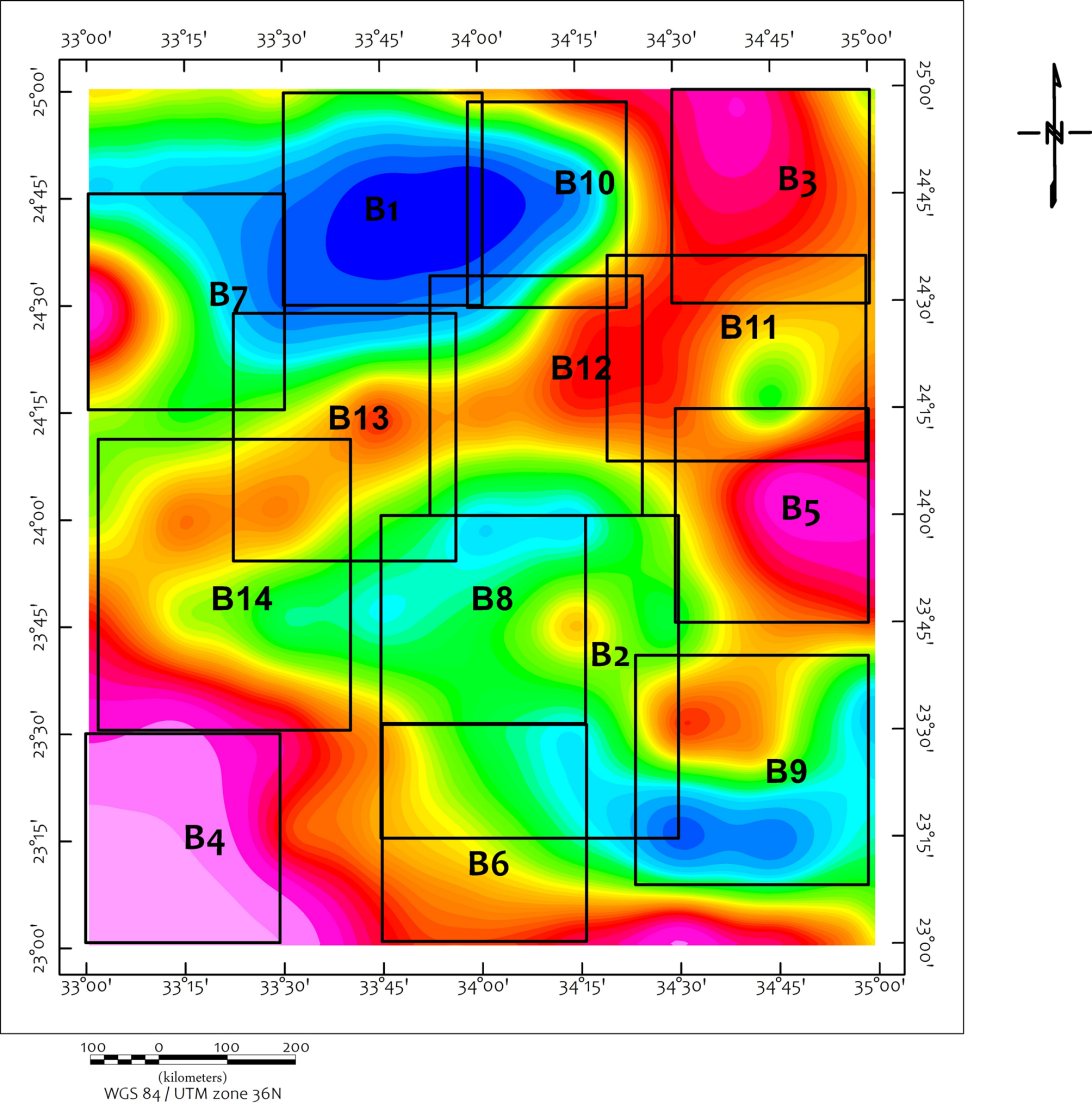
The RTP map is divided into 14 blocks to better understand the variation in depth to the basement.

**Fig. 13:**
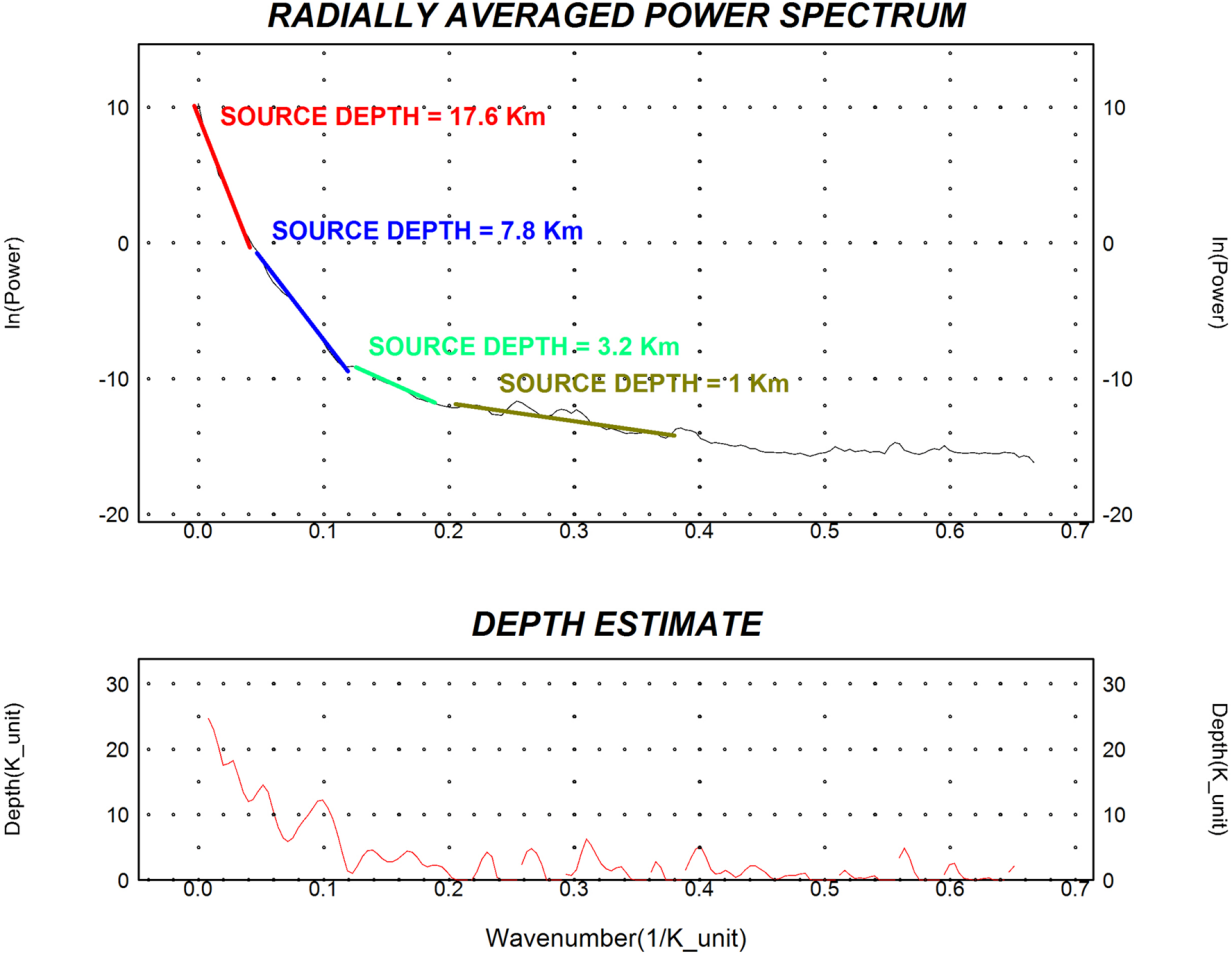
Power spectrum of the RTP data of the whole study area (Using Geosoft Oasis Montaj 2015 v. 8.4 software, https://www.seequent.com/help-support/oasis-montaj/).

To better understand the variation in basement depth across the region, the RTP data were divided into 14 interpretation blocks (Fig. 12), and power spectral analysis was applied to each block (Fig. 13).

**Fig. 14:**
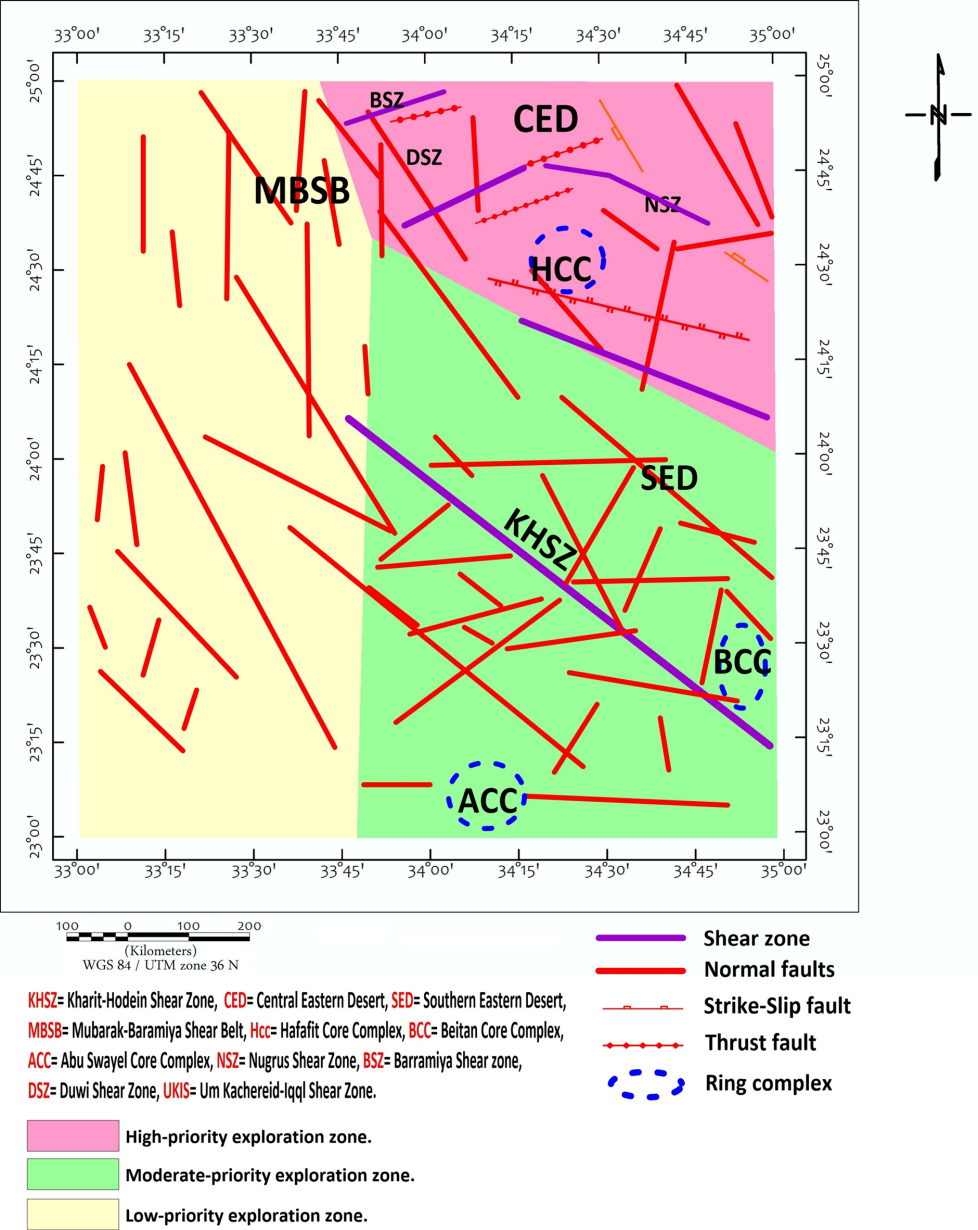
The final decision regarding the structural map of the study area can be a promising location for economic mineral mining in the study area (WGS 84/UTM Zone 36N), which shows major shear zones, faults, and ring complexes. The main structural elements include the Kharit–Hodein Shear Zone (KHSZ), Nugrus Shear Zone (NSZ), UKIS= Um Kachereid-Iqql Shear Zone, Barramiya Shear Zone (BSZ), and Duwi Shear Zone (DSZ). The map also delineates core complexes such as Hafafit (HCC), Beitan (BCC), and Abu Swayel (ACC), with fault types distinguished as normal, strike-slip, and thrust faults. (Using Geosoft Oasis Montaj 2015 v. 8.4 software, https://www.seequent.com/help-support/oasis-montaj/).

Euler deconvolution and power spectral analysis provide depth estimates to clusters of magnetic sources across the study area, with deeper sources ranging between approximately 7-18 km (Table 1, Fig. 14). These estimates carry quantified uncertainties: spectral analysis yields average depths to source ensemble centroids with uncertainties of ±15-20%, while Euler solutions are sensitive to window size and structural index selection, carrying uncertainties of approximately ±10-15%. The maximum estimated depth of ~18 km in Block 1 therefore, represents the approximate centroid depth of a magnetic source distribution, a cluster of sources contributing to the observed anomaly, rather than the top of a discrete geological body. These depth ranges are broadly consistent with regional crustal thickness estimates from seismic studies (e.g.^83^ but should not be directly linked to specific mineral systems without supporting evidence.

**Table 1:** The results of the depth to spectral analysis applied to the 14 blocks as well as the whole study area (Using Geosoft Oasis Montaj 2015 v. 8.4 software, https://www.seequent.com/help-support/oasis-montaj/).

Block No.	Deep source			Shallow source		
	Deep source (km)	Deep uncertainty (±%)	Deep depth range (km)	Shallow source (km)	Shallow sncertainty (±%)	Shallow depth range (km)
B. 1	18.0	15	15.3 – 20.7	4.7	12	4.1 – 5.3
B. 2	7.0	18	5.7 – 8.3	1.9	14	1.6 – 2.2
B. 3	7.9	16	6.6 – 9.2	4.3	11	3.8 – 4.8
B. 4	7.9	17	6.6 – 9.2	4.7	13	4.1 – 5.3
B. 5	9.4	15	8.0 – 10.8	5.0	10	4.5 – 5.5
B. 6	9.4	19	7.6 – 11.2	4.0	15	3.4 – 4.6
B. 7	8.2	16	6.9 – 9.5	4.3	12	3.8 – 4.8
B. 8	8.0	18	6.6 – 9.4	3.5	14	3.0 – 4.0
B. 9	10.0	15	8.5 – 11.5	4.0	11	3.6 – 4.4
B. 10	11.0	17	9.1 – 12.9	3.0	13	2.6 – 3.4
B. 11	10.0	16	8.4 – 11.6	3.6	12	3.2 – 4.0
B. 12	14.0	15	11.9 – 16.1	4.0	10	3.6 – 4.4
B. 13	15.0	18	12.3 – 17.7	5.5^a^	14	4.7 – 6.3
				(2.0)^a^	(15)	(1.7 – 2.3)
B. 14	11.0	17	9.1 – 12.9	7.0^a^	13	6.1 – 7.9
				(3.5)^a^	(14)	(3.0 – 4.0)
Full Area	**17.59**	**16**	**14.8 – 20.4**	**7.8**^**b**^	**15**	**6.6 – 9.0**
				**3.2**^**b**^	**12**	**2.8 – 3.6**
				**1.0**^**b**^	**14**	**0.9 – 1.1**

(^a^)For Blocks 13 and 14, two shallow magnetic source layers were resolved, representing distinct magnetic interfaces (e.g., shallow intrusions and near-surface contacts). The values in parentheses indicate the secondary shallow source.(^b^)For the Full Area, spectral analysis resolved three magnetic source ensembles: a deep source (~17.6 km), an intermediate source (~7.8 km), and two shallow sources (~3.2 km and ~1.0 km). The 1.0 km source likely corresponds to near-surface weathering or shallow magnetic bodies.All depth ranges are calculated as Depth ± (Depth × Uncertainty/100). Uncertainties are based on the method used: spectral analysis (deep sources) ±15–20%; Euler deconvolution (shallow sources) ±10–15%, consistent with the ranges discussed in Section "Results" (Limitations).

To assess the robustness of Euler solutions and determine the most appropriate structural index (SI) for the study area, sensitivity analysis was performed using SI values ranging from 0 to 3. Solutions derived with SI=0, corresponding to a contact/fault model, exhibit the highest spatial coherence with mapped fault systems and structural discontinuities, confirming their suitability for delineating fault-controlled contacts. Solutions derived from SI=1, representing dyke or thin-sheet models, show clustering near circular magnetic anomalies, suggesting the presence of dyke-like or intrusive bodies. In contrast, SI=2 solutions (cylindrical bodies) are more scattered with limited spatial coherence, while SI=3 solutions (spherical bodies) are relatively sparse. Based on these observations, SI=0 was adopted for the final structural interpretation due to its strong agreement with known tectonic trends, while SI=1 and SI=3 solutions were retained to assist in identifying potential intrusive sources. To enhance reliability, depth solutions with uncertainties exceeding 15% were excluded from the final interpretation. The consistent clustering of solutions across multiple SI values supports the interpretation that the subsurface architecture comprises both linear fault-bounded structures and deeper intrusive bodies, thereby providing a coherent model of structural geometry and depth variability within the study area^84^.

Results (Table 1, Fig. 14) indicate that deeper magnetic source clusters range between approximately 7 and 18 km, with the deepest values recorded in Block 1 (18 km centroid depth) and the full-area average reaching approximately 17.59 km. Shallower source clusters appear predominantly at depths between 3 and 5 km. These findings are in agreement with spectral analysis trends and reinforce the significant crustal heterogeneity of the region. The maximum estimated depth correlates spatially with the Kharit Basin depocenter, which is bounded by NW-striking faults associated with the KHSZ. The delineated shear zones, faults, and core complexes represent structural features that may have influenced fluid migration pathways; their interpretation is therefore relevant for identifying areas that may warrant further investigation for mineralization potential (Fig. 14).

Collectively, the integration of aeromagnetic data, structural filters, and depth estimation techniques reveals several geophysically distinct regions characterized by structural complexity and elevated magnetic anomaly intensities. The Wadi Kharit area, in particular, exhibits a convergence of structural complexity, magnetic anomaly intensity, and patterns suggestive of intrusive bodies, positioning it as an area of interest for further investigation. Furthermore, the alignment of regional magnetic trends with mapped lineaments and rift-related faults supports the interpretation of a structurally controlled setting favorable for fluid circulation and potential mineralization, though confirmation requires ground-based investigation.

## Discussion

The southern Eastern Desert of Egypt has emerged as a focal point for multidisciplinary investigations into hydrothermal and intrusion-related mineralization. Our analysis of airborne magnetic data across this region compels a re-evaluation of the structural architecture controlling known mineral occurrences and, more importantly, identifies specific, high-priority targets for exploration. The interpreted magnetic anomalies do not merely represent abstract geophysical features; they provide a compellingly direct image of a deeply structured crust, where the interplay between major shear zones and buried intrusions creates a highly prospective metallogenic framework.

Critically, these anomalies are not randomly distributed. Several of the most intense magnetic signatures align with remarkable fidelity along the NW- to NNW-trending sinistral strike-slip faults that constitute the regional tectonic grain. The Kharit–Hodein Megashear Zone (KHSZ), a ~100 km-long ductile shear zone, is not just a geological boundary on a map. Our data show it as a profound crustal discontinuity, its pronounced displacement and deformation fabrics exerting a first-order control on the development of adjacent basins and acting as the primary conduit for deep-seated magmas and hydrothermal fluids. Similarly, the magnetic fabric around the Nugrus Shear Zone vividly demarcates the complex boundary between the Wadi Ghadir ophiolites and the Hafafit Core Complex (HCC), a terrain whose subsurface complexity is now revealed by deep-seated magnetic anomalies extending south of the HCC, suggestive of buried batholiths or E–W trending igneous bodies.

The strongest evidence for a linked structural-magmatic mineral system, however, concentrates in the Wadi Kharit region. Here, the coincidence of intense magnetic signatures, two identified intrusions, and a dense structural network is unequivocal. The application of advanced filters, CET porphyry analysis, CET-GA, and SED, collectively delineates a coherent subsurface architecture. Crucially, the Euler depth solutions validate the significance of this zone, calculating basement contact depths reaching up to 18 km. This depth highlights the structural maturity of the conduit system and suggests a deeply rooted, long-lived magmatic-hydrothermal system, a key ingredient for significant mineral endowment.

This geophysically defined structural framework directly contextualizes and refines previous surface-based observations. Earlier studies have firmly established that orogenic and intrusion-related gold and copper occurrences in the region are spatially linked to granitoids and shear zones^50^. Detailed work in districts like Hamash confirmed quartz-vein hosted Au-Cu mineralization with alteration assemblages (sericitization, silicification) pointing to a magmatic-hydrothermal origin^35,85^. Our magnetic data now provide the missing link: they demonstrate that these surface occurrences are not isolated phenomena but are surface expressions of a deep-seated, district-scale plumbing system. The NW–NNW and N–S orientations dominant in our rose-diagram and lineament analyses^50,86,87^ are shown to be not merely surface fractures but the surface traces of deep, magnetic crustal flaws. The structural model derived from case studies at Hamash, Barramiya, and Um Garayat, where brittle–ductile shear zones focus fluid flow^42^, is therefore not just a local explanation but a regional paradigm, powerfully imaged in our geophysical data.

The economic implications of these findings are substantial. The convergence of a favorable structural setting, robust and deep-seated magnetic anomalies, and relatively accessible terrain elevates the Wadi Kharit corridor and the flanks of the Hafafit Core Complex from areas of academic interest to strategic exploration targets. The structural concentration, high magnetic intensities, and multiple coinciding geophysical indicators define a high-potential zone that warrants immediate ground-based follow-up. This interpretation is robustly supported by analogues across the broader Arabian–Nubian Shield. The porphyry Cu–Au system at Jebel Ohier in Sudan^88^, the REE-enriched A-type granites of the Igla Ahmr area controlled by Najd faulting^89^, and the broader recognition of shear zones as first-order controls on orogenic gold and VMS systems^47^ all validate the predictive power of our model. Our analysis suggests that the Wadi Kharit area shares the fundamental geological ingredients of these productive provinces.

It is imperative to state clearly that these interpretations are derived entirely from airborne magnetic data, in the absence of direct field verification. This study is therefore a predictive geophysical framework. The mapped structures and inferred mineralized zones are not confirmed geological observations but data-supported hypotheses. This approach, however, is its strength. By integrating multiple independent filters, tilt derivative, vertical and horizontal gradients, CET, SED, and Euler deconvolution, we have built a coherent and internally consistent model of the subsurface that aligns with known regional trends. This geophysical foundation serves its purpose: it is a highly efficient first-pass tool, reducing the footprint for expensive and time-consuming initial fieldwork. The next logical and necessary step is to move from prediction to validation. We strongly recommend targeted ground-based geophysical and geochemical investigations, specifically in the Wadi Kharit corridor and the structurally complex zones adjacent to the Hafafit Core Complex, to verify these interpretations, refine the subsurface model, and ultimately test for the presence of economic mineralization.

## Limitations of the study

Several limitations inherent to this study must be acknowledged when interpreting the results. First, the airborne magnetic data comprises a regional 2-arc-minute grid (~3.7 km cell size) acquired at approximately 4 km altitude, which limits sensitivity to shallow, small-scale features; while enhancement filters improve interpretability, they do not increase the intrinsic resolution of the original survey. Second, potential field interpretations are governed by the non-uniqueness principle, wherein multiple source geometries and susceptibility distributions can produce mathematically identical magnetic anomalies. Although the integration of independent filters (TDR, VD, HG, CET, SED, Euler) reduces interpretational ambiguity by requiring consistency across methods, it cannot eliminate the fundamental non-uniqueness inherent to potential field geophysics. Third, a critical distinction must be made between magnetic sources and actual lithologies; anomalies interpreted as ophiolitic assemblages, granitic intrusions, or interpreted as shear zones represent geophysically defined bodies whose lithological composition remains inferred until validated by direct observation. Fourth, all interpretations derive solely from magnetic data processing without direct field verification; therefore, structural lineaments, lithological correlations, and prospectivity zones remain predictive and require systematic ground-truthing through geological mapping, rock sampling, and geochemical analysis to confirm interpreted features. Fifth, depth estimates carry substantial uncertainty: spectral analysis yields average depths to magnetic source ensembles with uncertainties of approximately ±15-20%, while Euler solutions are parameter-sensitive with uncertainties of ±10-15% depending on window size and structural index selection. Consequently, the ~18 km maximum depth represents an approximate centroid of a magnetic source distribution rather than a precise contact depth. Sixth, CET porphyry signatures, circular magnetic patterns suggestive of intrusive centers, must be interpreted cautiously, as alternative explanations such as mafic complexes, magnetic sedimentary basins, or structural interference patterns are equally plausible without supporting geochemical, alteration, or petrophysical data.

Collectively, these limitations establish this work as a predictive framework requiring systematic ground validation before any economic conclusions can be drawn. All interpretations presented herein should therefore be regarded as inferred, interpreted, or consistent with specific geological models, not as definitive identifications of mineral deposits or lithological units.

## Conclusion

This study processed and interpreted regional airborne magnetic data from the southern Eastern Desert of Egypt to delineate structural controls on potential mineralization and identify prospective zones for gold, copper, and rare earth element exploration. Multiple enhancement filters, including tilt derivative, vertical and horizontal gradients, CET porphyry analysis, source edge detection, Euler deconvolution, and spectral analysis were integrated to extract maximum structural information from the magnetic data and estimate depths to magnetic sources.

The interpretation reveals a structurally complex terrain dominated by NW-NNW sinistral strike-slip faults, including the Kharit-Hodein Megashear Zone and Nugrus Shear Zone, which control the regional tectonic framework. Depth estimates indicate magnetic source clusters extending to approximately 18 km, suggesting crustal-scale structures that may have focused hydrothermal fluid flow. Integration of magnetic anomalies, structural lineaments, and porphyry signatures delineates the Wadi Kharit depocenter and Hafafit Core Complex margins as priority exploration targets. These findings provide a data-driven predictive framework for subsequent ground investigation, demonstrating the value of airborne magnetic surveys as a cost-effective first step in mineral exploration within complex shield terrains. Future work should prioritize targeted ground-based geophysical and geochemical surveys within these identified zones to validate interpreted structures, test for potential mineralization, and evaluate economic potential.

## Supplementary Information

Supplementary Information

## Data Availability

The airborne magnetic survey data used in this study are available from the Geological Survey of Egypt (GSE) upon reasonable request and with permission from the relevant authorities. Derived data supporting the findings of this study, including processed magnetic maps and structural interpretations, are available from the corresponding author upon request.
