# Supplementary material for: Delineation of shear zone-hosted mineral targets within the Arabian-Nubian Shield, Egypt, using aeromagnetic data
Source: Sci Rep. 2026 Apr 17;16:12702. doi: 10.1038/s41598-026-45708-6 (PMC13090388; doi:10.1038/s41598-026-45708-6)
Supplement: Supplementary file 1 — Supplementary Information [file 41598_2026_45708_MOESM1_ESM.docx]

**Supplementary Material**

1. **The tilt derivative (TDR)**

The Time-Domain Tilt Derivative (TDR) is a technique applied to spatial magnetic geophysical data to investigate shallow basement structures, identify mineralized zones, and map the boundaries of source bodies. Originally proposed by **^92^** and subsequently enhanced by **^93^**, this tilt filter has seen continued development by researchers such as **^94,95^**,and **^96^**, owing to its practical and conceptual simplicity. The TDR is computed by taking the arctangent of the ratio between the vertical (VDR) and horizontal (THDR) derivatives:

${TDR= tan}^{-1}\frac{VDR}{THDR}$ (1)

In more specific mathematical terms, the TDR is given by:

i.e.: $TDR={tan}^{-1}\frac{\left( \frac{\partial f}{\partial z} \right)}{\sqrt{{(\partial f/\partial x)}^{2}+(\partial f/{(\partial f/\partial y)}^{2}}}$ (2)

where *f* represents the magnetic or gravity field, and (δƒ/δx, δƒ/δy), and (δƒ/δz) are the field’s first derivatives along the x, y, and z directions, respectively. This approach is particularly effective in interpreting potential field data and has demonstrated valuable applications across different geological settings.

1. **The Vertical Derivative Filter**

The vertical derivative filter is a widely used enhancement technique in the processing of potential field data, especially in magnetic and gravity surveys. It enhances short-wavelength, high-frequency components of the data, making it particularly effective for delineating shallow geological features such as faults, contacts, and intrusions, while suppressing deeper, regional signals.

Mathematically, the vertical derivative of a potential field $f (x,y,z)$ is given by:

$$VDF= \frac{\partial f}{\partial z}\ldots\ldots\ldots\ldots\ldots\ldots\ldots\ldots\ldots\ldots\ldots\ldots\ldots\ldots(3)$$

In the frequency domain, the vertical derivative is implemented by multiplying the Fourier transform of the data by the vertical wavenumber k_z_​, which increases with frequency. The general form in the Fourier domain is:

$$F \left( \frac{\partial f}{\partial z} \right)=k.F \left[ f\left( x,y \right) \right]\ldots\ldots\ldots\ldots\ldots\ldots\ldots\ldots\ldots\ldots\ldots\ldots\ldots. (4)$$

Where

$${K=sqrt (K}_{x}^{2}+ K_{y}^{2})\ldots\ldots\ldots\ldots\ldots\ldots\ldots\ldots\ldots\ldots\ldots\ldots\ldots\ldots\ldots.. (5)$$

is the radial wavenumber, and FFF denotes the Fourier transform.

While the vertical derivative significantly improves the visibility of shallow features, it can also amplify high-frequency noise, making data conditioning and careful interpretation essential. Despite this, it remains a key tool in structural mapping and mineral exploration.

1. **Horizontal Gradient Filter**

The horizontal gradient filter is a fundamental tool in potential field data interpretation, particularly for defining the lateral borderlines of subsurface structures. It enhances the edges of geological bodies by highlighting the rate of change of the field in the horizontal plane. This method is particularly useful in identifying contacts, faults, and other discontinuities where physical property contrasts exist **^65^**. The horizontal gradient magnitude (HGM) of a potential field $f \left( x,y \right)$such as magnetic or gravity data, is mathematically expressed as:

$$\left| \nabla hf \right|=sqrt {(\left( \partial f/\partial x \right)}^{2}+\left( \partial f/\partial y \right)^{2})\ldots\ldots\ldots\ldots\ldots\ldots\ldots\ldots\ldots\ldots\ldots\ldots\ldots\ldots.. (6)$$

Where $\partial f/\partial x$ and $\partial f/\partial y$ represent the partial derivatives of the field in the x and y directions, respectively. This formulation provides a scalar field that emphasizes zones of maximum horizontal rate of change, which often correlate with geological boundaries. One of the key advantages of the horizontal gradient is its relative insensitivity to the depth of the source, making it effective in mapping features across various depths with minimal assumptions about geometry.

1. **Theta Map (Tilt Angle) Filter**

The theta map filter, also identified as the tilt angle filter, is a scale-independent edge enhancement technique introduced by **^97^** for interpreting potential (magnetic and gravity anomalies) field data. It is particularly effective for delineating both shallow and deep sources, as it normalizes the vertical derivative by the horizontal gradient magnitude, making structural boundaries equally visible regardless of depth **^98^**. The filter produces a continuous map where the edges of source bodies appear as zero contours, while positive and negative values reflect the position and polarity of the anomaly. The tilt angle θ\thetaθ is defined as:

$$TM=\mathrm{acos} \left( \frac{HG}{\left| ASA \right|} \right)\ldots\ldots\ldots\ldots\ldots\ldots\ldots\ldots\ldots\ldots\ldots\ldots\ldots..(7)$$

The tilt angle effectively balances depth effects and enhances edge continuity, achieving it a powerful tool in structural mapping and mineral exploration. Its utility lies in its ability to trace subtle and deep-seated features without requiring prior knowledge of source geometry.

1. **Center for Exploration-Targeting Grid-Analysis (CET-GA) Technique**

The CET-GA Grid Analysis extension offers tools for Texture Analysis, Lineation Detection, Lineation Vectorization, and Structural Complexity, all aimed at identifying lineaments and determining promising regions for ore deposits **^74^**. This method enhances zones of discontinuity within potential field data, focusing on the variations of magnetic intensity to highlight structural features. By identifying complex texture zones in the magnetic data and locating axes of symmetry, this technique identifies linear disturbances in the signal that correlate with magnetic discontinuities linked to geological features like rock boundaries, linear structures, and intrusions.

Magnetic discontinuities are represented as skeletal structures by enhancing textures within the data, producing outputs that visualize each discontinuity zone as a series of line segments. These segments reflect directional shifts and offsets within the structural features. The CET-GA process typically includes the following steps:

1. Texture Enhancement: Identifies areas with intricate textures associated with magnetic discontinuities.
2. Phase Symmetry: Utilizes texture enhancement results to outline zones of lateral discontinuity **^75,79,80^**.
3. Structural Delineation: Converts these discontinuity zones into distinct, line-like structures using phase symmetry outputs **^81^**.

This structured approach is valuable for characterizing geological settings, especially in the context of mineral exploration.

1. **Source edge detection (SED)**

Source edge detection is a critical geophysical technique employed in research centers to delineate the boundaries of subsurface geological bodies based on potential field data, such as magnetics or gravity **^99^**. It enhances the interpretation of structural features by identifying sharp lateral contrasts in physical properties, which typically correspond to faults, lithological contacts, or intrusive margins. Methods such as the horizontal gradient, total horizontal derivative, and tilt angle are commonly applied to highlight these edges **^65,94,95^**. Accurate edge detection not only supports the interpretation of geological mapping but also supports mineral exploration by outlining zones of structural control and potential ore emplacement. This technique is particularly valuable in complex terrains where direct geological observations are limited.

1. **Center for Exploration-Targeting (CET) Porphyry Analysis**

Exploration-targeting porphyry analysis is a specialized geophysical and geological approach designed to identify and prioritize potential porphyry copper and gold systems, which are among the most economically significant mineral deposits globally. This analysis integrates multiple datasets such as magnetic, gravity, radiometric, and geological information to detect characteristic signatures of porphyry systems, including circular magnetic anomalies, central high amplitude zones, and alteration halos **^74,81^**. Advanced filters and algorithms, such as the CET Porphyry Analysis, enhance features like concentric zoning and radial structures that typify porphyry deposits. By systematically evaluating these indicators, research centers can reduce exploration risk, focus field efforts, and improve the efficiency of mineral discovery in complex terrains.

1. **Euler Deconvolution**

Euler deconvolution is an automated interpretation technique widely applied in potential field analysis to estimate the location and depth of subsurface sources. The method is based on Euler’s homogeneity equation, which relates the spatial derivatives of the observed field to the position of its causative source, assuming a predefined Structural Index (SI) that reflects the geometry of the source body **^100,101^**. In this study, Euler deconvolution was implemented using a 10 × 10 km moving window (approximately three times the grid cell size) with 50% overlap. The analysis was initially conducted with a fixed SI value of 0, appropriate for mapping geological contacts and fault boundaries characterized by abrupt contrasts in magnetic susceptibility, and subsequently repeated using SI values ranging from 0 to 3 to evaluate solution sensitivity. These values correspond to different source geometries, including contacts or faults (SI=0), dyke or sill-like bodies (SI=1), cylindrical sources (SI=2), and spherical bodies (SI=3).Top of FormBottom of Form

1. **Spectral Analysis**

Spectral analysis is a frequency-domain technique widely used in the interpretation of potential field data, particularly magnetic and gravity surveys. It involves decomposing the observed field into its frequency components using the Fourier Transform, allowing discrimination between sources at different depths based on their wavelength content. Shallow sources typically generate high-frequency anomalies, whereas deeper sources are associated with low-frequency signals **^99,101,102^**. By examining the logarithmic power spectrum, the depth to magnetic sources can be estimated from the slope of linear segments in a plot of log power versus wavenumber, where the slope is proportional to the source depth **^92^**.

In this study, power spectral analysis was applied to 14 square blocks, each measuring 50 × 50 km (approximately 13 × 13 grid cells), with a 25% overlap between adjacent blocks to ensure continuous spatial coverage. For each block, the radially averaged power spectrum was calculated, and depth estimates were derived from the linear segments of the log power–wavenumber plots using the relation slope = −4π × depth. This approach enables the characterization of both regional (deep) and residual (shallow) source components and provides an efficient preliminary assessment of depth distribution before the application of spatial-domain filtering or inversion methods. The resulting depth estimates for the individual blocks, as well as for the entire study area, are presented in Table (1) (processed using Geosoft Oasis Montaj 2015, version 8.4; <https://www.seequent.com/help-support/oasis-montaj/>).
